# TNF-α-308G/A Polymorphism Contributes to Obstructive Sleep Apnea Syndrome Risk: Evidence Based on 10 Case-Control Studies

**DOI:** 10.1371/journal.pone.0106183

**Published:** 2014-09-05

**Authors:** Yanping Wu, Chao Cao, Yinfang Wu, Chao Zhang, Chen Zhu, Songmin Ying, Zhihua Chen, Huahao Shen, Wen Li

**Affiliations:** 1 Department of Respiratory and Critical Care Medicine, Second Affiliated Hospital, School of Medicine, Zhejiang University, Hangzhou, China; 2 Department of Respiratory Medicine, Affiliated Hospital of School of Medicine, Ningbo University, Ningbo, China; The University of Hong Kong, Hong Kong

## Abstract

**Objective:**

The aim of our study was to investigate the association between the *TNF-α-308G/A* polymorphism and obstructive sleep apnea syndrome (OSAS).

**Method:**

The Medline, Web of Science, EMBASE, Chinese National Knowledge Infrastructure (CNKI), and Cochrane Central Register of Controlled Trials were searched. Pooled odds ratios (ORs) and 95% confidence intervals (CIs) were calculated to study *TNF-α-308G/A* polymorphism and risk of OSAS.

**Result:**

10 case-control studies were included in our meta-analysis. The results from our study showed that the *TNF-α-308G/A* polymorphism was significantly associated with risk of OSAS (A *vs.* G: OR = 1.67, 95% CI = 1.43–1.95). In the subgroup analysis by ethnicity, the statistical similar results were observed both in European (A *vs.* G: OR = 1.68, 95% CI = 1.35–2.08) and Asian population (A *vs.* G: OR = 2.02, 95% CI = 1.50–2.71). When stratified by age, a significantly increased risk was observed in adult carries A allele compared with G allele (OR = 1.79, 95% CI = 1.50–2.13), whereas no association was found in children (OR = 1.09, 95% CI = 0.70–1.69).

**Conclusion:**

Our study suggested that the *TNF-α- 308G/A* polymorphism contributed to the susceptibility to the risk of OSAS. Additional well-designed large studies are needed to validate our findings.

## Introduction

Obstructive sleep apnea syndrome (OSAS) is a common sleep disorder characterized by repetitive partial or complete obstruction of the upper respiratory tract during sleep, resulting in apnea or hypopnea [1]. Due to obesity and aging population, the OSAS has undergone an increasing prevalence all over the world. It was reported that more than 5% of the general population has been affected [2]. According to the National Sleep Foundation (NSF) Sleep in America, there were 1/4 Americans at high risk of suffering sleep apnea on the basis of the Berlin Questionnaire [3]. OSAS has been reported to be associated with various health related consequences, including cardiovascular disease, hypertension, stroke, insulin resistance and all-cause mortality [4]. OSAS represents a vital public health concern and should be given much more attention because of the high prevalence and its enormous negative consequences. In consequence, improving our understanding of the pathogenesis of OSAS is essential for the development of effective and safe therapies.

Tumor necrosis factor (TNF)-α, a member of the TNF/TNFR cytokine family, is an intercellular communicating molecule involved in a wide variety of human diseases. Krueger et al. [5] has pointed out that TNF-α is one of the most important pleiotropic proinflammatory cytokines involved in sleep regulation. Raised levels of circulating TNF-α in patients with OSAS have been reported in previous studies [6]–[8]. The synthesis of TNF-α has been suggested to be mostly regulated at the transcriptional level [9]. The DNA variations in the promoter region of the *TNF-α* gene may directly influence the transcription of the *TNF* gene. The *TNF-α* gene is located within the highly polymorphic major histocompatibility complex (MHC) region on the short arm of chromosome 6p21.3 [10]. Several polymorphisms in the promoter region of the *TNF-α* gene have been identified. Among which, polymorphism at position −308 in the promoter region, consisting of a guanine (G) to adenine (A) substitution, has been reported to be associated with increased production of TNF-α levels both *in vitro* and *in vivo* [11], [12]. There is evidence that A allele is associated with increased level of TNF-α in plasma compared with G allele [13]. As the *TNF-α-308G/A* polymorphism is strongly associated with circulating TNF-α concentrations, it is assumed that it might be closely related to the OSAS risk. To investigate a possible association between *TNF-α* polymorphism and risk of OSAS, we conducted a meta-analysis from all available relevant studies.

## Materials and Methods

### Literature search strategy and eligibility criteria

The Medline, Web of Science, EMBASE, Chinese National Knowledge Infrastructure (CNKI) and Cochrane Central Register of Controlled Trials were searched. A broad search strategy was used for optimum sensitivity. Using Medical Subject Headings (MeSH) and text words, we adopted the following terms to search the databases: (“obstructive sleep apnea–hypopnea syndrome” OR “obstructive sleep apnea” OR “sleep apnea” OR “apnea” OR “OSAS” OR “OSA”) AND (“single nucleotide polymorphisms” OR “SNP” OR “polymorphism” OR “gene variant” OR “mutation”) AND (“tumor necrosis factor α” OR “tumor necrosis factor-α” OR “tumor necrosis factor” OR “TNF-α” OR “TNF”). The search was restricted to humans. Titles and abstracts were screened up to 31 March 2014 were retrieved. Articles were screened at the title and abstract phase by two authors (Yanping Wu and Chao Cao).

### Inclusion and exclusion criteria

Inclusion criteria for studies included: (a) evaluation of the relationship between *TNF-α- 308G/A* polymorphism and OSAS susceptibility; (b) case–control study; (c) validated genotyping methods were used; (d) detailed genotype frequencies in cases and controls for the calculation. Major reasons for exclusion of studies were: (a) no control group, case reports or review articles; (b) duplicate data; (c) enrolled patients were using TNF inhibitors.

### Data extraction and quality assessment

Information was carefully extracted from all eligible studies independently by two of the authors, according to the inclusion criteria listed above. From each study, data on general study characteristics was extracted: first author's surname, year of publication, ethnic descent of the study population (European, Asian or mixed), genotyping methods, numbers of eligible cases and controls, genotype distributions in cases and controls and available subgroups. Disagreements were resolved by discussion and consensus.

The Newcastle-Ottawa Scale (NOS), which was developed to assess the quality of non-randomised studies, was performed to assess the quality of included studies [14]. The system included three broad perspectives: the selection of the study groups; the comparability of the groups; and the ascertainment of the outcome. And the scores of 0–3, 4–6, and 7–9 indicated low, moderate, and high quality of studies, respectively.

### Statistical analysis

All analyses were performed on Review Manager version 5.0.16 and Stata version 12.0. The Hardy-Weinberg equilibrium (HWE) was utilized to compare the observed genotype frequencies with expected genotype frequencies in controls. The association between risk of OSAS and *TNF-α-308G/A* polymorphism was estimated for each study using the odds ratio (OR) and 95% confidence interval (CI). Between-study heterogeneity was assessed with the χ^2^-based Q statistical test [15]. We also used the statistic of *I^2^* to efficiently test for the heterogeneity, with *I^2^*<25%, 25–75% and >75% to represent low, moderate and high degree of inconsistency, respectively [16], [17]. When *P*>0.1 or *I^2^*<50%, indicating a lack of heterogeneity, and for these analyses, the fixed-effect model was used; otherwise, the random-effect model was applied [16]–[19]. The significance of the combined OR was determined by the Z-test, in which *P*<0.05 was considered significant. Effects were tested in models as follows: allele comparison (A vs. G), homozygote comparison (GG vs. AA), heterozygote comparison (AG vs. AA), dominant model (AG+GG vs. AA), and recessive model (GG vs. AA+AG). Stratified analysis was performed by ethnicity and age. Sensitivity analysis was performed to evaluate the effect of each individual study on the pooled ORs. Funnel plots and Egger's linear regression test were used to evaluate the potential publication bias [20].

## Results

### Characteristics of studies

**Figure 1:**
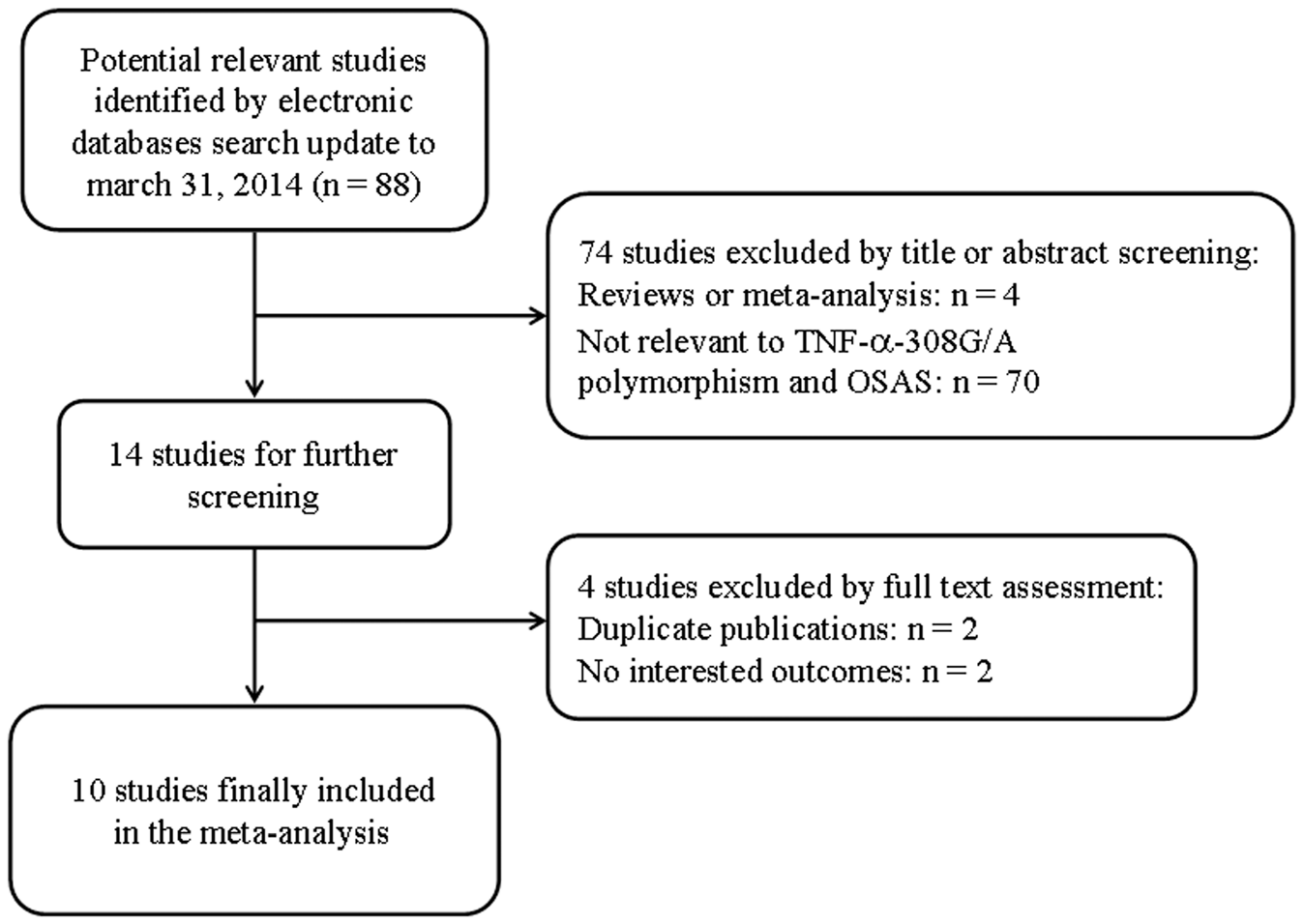
Flow chart of literature search and study selection.

The search strategy retrieved 88 potentially relevant studies. After initial search by the titles and abstracts, 74 studies were excluded. Among the excluded studies, 4 were reviews or meta-analysis and 70 were not relevant to *TNF-α-308G/A* polymorphism and OSAS. By further full text screening, 2 studies were duplicate publications and 2 had no interested outcomes. Finally, 10 full-text articles, with a total of 1522 OSAS cases and 1234 controls, which definitely addressed the association between *TNF-α-308G/A* polymorphism and OSAS risk were enrolled in our study [21]-[30] (Figure 1). According to the NOS quality assessment, all included studies achieved a score of moderate to high quality (table S1). The genotype distribution in each study was extracted and detailed information in one study was referred to published data. Of all eligible studies for *TNF-α-308G/A* polymorphism, four were performed in European population [23], [24], [27], [30], five in Asian descent [21], [22], [26], [28], [29] and one in mixed populations (European and Asian descents) [25]. In terms of age, nine studies were conducted in adults [21]–[24], [26]–[30] and one in children [25]. Polymerase chain reaction-restriction fragment length polymorphism (PCR-RFLP) [22]–[24], [26]–[28] and TaqMan assay [21], [25], [29], [30] were used to analyze the genotyping. The genotype distributions of all control samples except one study were in HWE [26]. The general characteristics and genotype information of each study were summarized in Table 1 and Table 2.

**Table 1:** Study characteristics from published studies on the relation of the TNF-α-308G/A polymorphism to OSAS risk in this meta-analysis.

Author	Year	Country	Ethnicity	Cases	Controls	Male (%)		Age, Mean(SD) [Range], y		BMI(kg/m^2^)		Source of control	Controls matched for	Methods
						Cases	Controls	Cases	Controls	Cases	Controls			
Popko *et al* ^27^	2008	Poland	European	102	77	72.6	50.7	[21.0–77.0]	[17.0–65.0]	NA	NA	Population based	BMI	PCR-RFLP
Khalyfa *et al* ^25^	2011	United States	mixed	138	151	50	50	(7.2±0.2)	(7.2±0.3)	NA	NA	Population based	age, sex, ethnicity and BMI	TaqMan
Riha *et al* ^30^	2005	United Kingdom	European	103	190	80.6	NA	(52.0±9.0)	NA	30.0±6.0	NA	Population based	not describled	TaqMan
Liu *et al* ^28^	2006	China	Asian	76	42	88.2	88.1	(44.3±9.8)	(41.7±10.1)	26.3±3.5	25.7±3.3	Hospital based	BMI	PCR-RFLP
Karkucak *et al* ^23^	2012	Turkish	European	69	42	75.4	69.1	(49.8±11.3)	(50.6±10.7)	31.1±4.1	28.5±4.3	Hospital based	age, gender and BMI	PCR-RFLP
Almpanidou *et al* ^24^	2012	Greece	European	220	319	90	87.1	(51.0±12.4)	(50.6±13.8)	31.4±5.2	29.6±4.3	Population based	age, race and gender	PCR-RFLP
Li *et al* ^21^	2013	China	Asian	155	100	100	100	(45.0±9.0)	(46.3±8.0)	29.4±2.1	24.1±2.3	Population based	not describled	TaqMan
Bhushan *et al* ^26^	2009	Indians	Asian	104	103	80.8	63.1	(46.2±10.7)	(44.0±10.0)	31.5±4.3	30.9±4.3	Hospital based	age and BMI	PCR-RFLP
Guan *et al* ^22^	2013	China	Asian	531	162	85.1	77.8	(43.6±11.7)	(42.6±13.1)	27.4±3.4	26.9±3.7	Hospital based	age, gender and BMI	PCR-RFLP
Li *et al* ^29^	2006	China	Asian	24	48	91.7	87.5	(39.7±7.9)	(38.3±9.2)	30.6±4.6	29.7±4.6	Hospital based	age, race, gender and BMI	TaqMan

NA: Not applicable; BMI: Body mass index; PCR-RFLP: Polymerase chain reaction–restriction fragment length polymorphism.

**Table 2:** Genotype distribution in included studies.

Author	Case			Control			G allele frequency (%)	HWE(P)
	AA	AG	GG	AA	AG	GG		
Popko *et al* ^27^	0	29	73	0	18	59	88.3	0.25
Khalyfa *et al* ^25^	7	33	98	5	38	108	84.1	0.47
Riha *et al* ^30^	7	44	52	8	52	130	82.1	0.34
Liu *et al* ^28^	8	23	45	1	6	35	90.5	0.27
Karkucak *et al* ^23^	0	18	51	0	9	33	89.3	0.44
Almpanidou *et al* ^24^	19	83	118	14	84	221	82.4	0.11
Li *et al* ^21^	0	18	137	0	12	88	94.0	0.52
Bhushan *et al* ^26^	8	22	74	3	10	90	92.2	<0.01
Guan *et al* ^22^	6	95	430	1	18	143	93.8	0.60
Li *et al* ^29^	3	10	11	1	12	35	85.4	0.98

HWE: Hardy-Weinberg equilibrium.

### Quantitative synthesis

**Figure 2:**
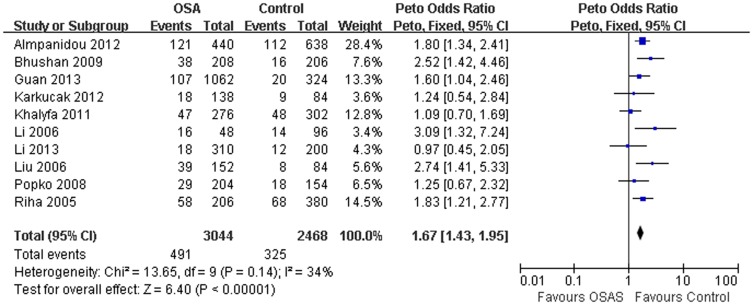
Meta-analysis with a fixed model for the ORs of OSAS risk associated with *TNF-α-308G/A* polymorphism (A vs. G).

**Figure 3:**
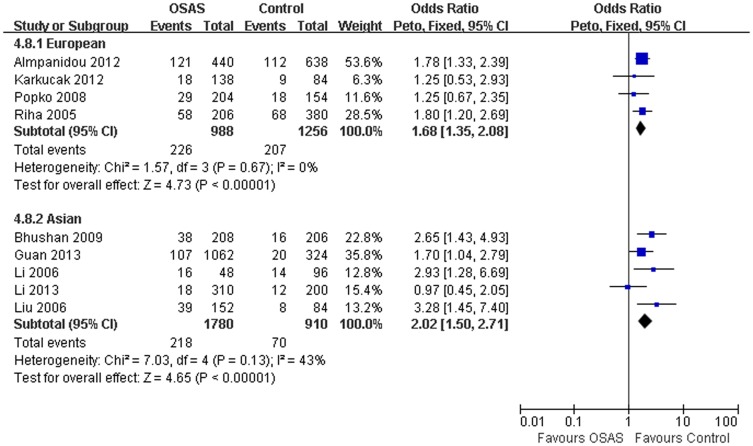
Meta-analysis with a fixed model for the ORs of OSAS risk in subgroup analysis by ethnicity (A vs. G).

Our study suggested a significant association of the *TNFα-308G/A* polymorphism with OSAS risk (A *vs.* G: OR = 1.67, 95% CI = 1.43–1.95, P<0.00001; homozygote comparison: OR = 2.62, 95% CI = 1.68–4.09, P<0.0001; dominant model: OR = 1.74, 95% CI = 1.36–2.22, P<0.00001; recessive model: OR = 2.15, 95% CI = 1.39–3.32, P* = *0.0006, Table 3; Figure 2). However, no significant association between this polymorphism and OSAS risk was observed in heterozygote comparison (OR = 1.38, 95% CI = 0.86–2.21, P = 0.18). In the subgroup analysis by ethnicity, similar results were observed in European (A *vs.* G: OR = 1.68, 95% CI = 1.35–2.08, P<0.00001) and Asian population (A *vs.* G: OR = 2.02, 95% CI = 1.50–2.71, P<0.00001; Figure 3). When stratified according to age, significant association between the *TNFα-308G/A* polymorphism and risk of OSAS was observed in adults (A *vs.* G: OR = 1.79, 95% CI = 1.50–2.13, P < 0.00001), but not for children (A *vs.* G: OR = 1.09, 95% CI = 0.70–1.69, P = 0.71).

**Table 3:** Stratified analysis of the *TNF-α-308G/A* polymorphism on OSAS risk.

Variables	No.^a^	Cases/Controls	A vs G			AA vs GG			AA vs AG			AA vs AG+GG			AA+AG vs GG		
			OR(95%CI)	*P* b	*P*	OR(95%CI)	*P* b	*P*	OR(95%CI)	*P* b	*P*	OR(95%CI)	*P* b	*P*	OR(95%CI)	*P* b	*P*
**Total**	10	1522/1234	1.67[1.43,1.95]	0.14	0.00	2.62[1.68,4.09]	0.79	0.00	1.38[0.86,2.21]	0.98	0.18	2.15[1.39,3.32]	0.91	0.00	1.74[1.36,2.22]	0.14	0.00
**Ethnicities**																	
European	4	494/628	1.68[1.35,2.08]	0.67	0.00	2.42[1.33,4.41]	0.82	0.00	1.25[0.67,2.33]	0.67	0.48	1.92[1.07,3.46]	0.74	0.03	1.83[1.41,2.36]	0.57	0.00
Asian	5	890/455	2.02[1.50,2.71]	0.13	0.00	3.95[1.56,10.00]	0.76	0.00	1.60[0.59,4.33]	0.87	0.36	3.28[1.30,8.26]	0.84	0.01	2.01[1.45,2.80]	0.16	0.00
mixed	1	138/151	1.09[0.70,1.69]	NA	0.71	1.54[0.47,5.02]	NA	0.47	1.61[0.47,5.56]	NA	0.45	1.56[0.48,5.04]	NA	0.46	1.03[0.62,1.71]	NA	0.92
**Age**																	
Adults	9	1384/1083	1.79[1.50,2.13]	0.30	0.00	2.80[1.69,4.63]	0.85	0.00	1.34[0.79,2.27]	0.96	0.27	2.24[1.37,3.69]	0.87	0.00	1.89[1.55,2.32]	0.36	0.00
Children	1	138/151	1.09[0.70,1.69]	NA	0.71	1.54[0.47,5.02]	NA	0.47	1.61[0.47,5.56]	NA	0.45	1.56[0.48,5.04]	NA	0.46	1.03[0.62,1.71]	NA	0.92

a number of studies.

b *P* value of *Q*-test for heterogeneity test.

0.00 means value<0.01.

### Sensitivity analysis

Sensitivity analysis was carried out to assess the influence of each individual study on the pooled ORs. By removing each study, the pooled ORs were not altered significantly, which indicated that no individual study significantly affected the pooled results. When one HWE-violating study was excluded, the corresponding pooled ORs were still meaningful, showing the results were robust (OR = 1.62, 95% CI = 1.37–1.91, P<0.00001).

### Publication bias

**Figure 4:**
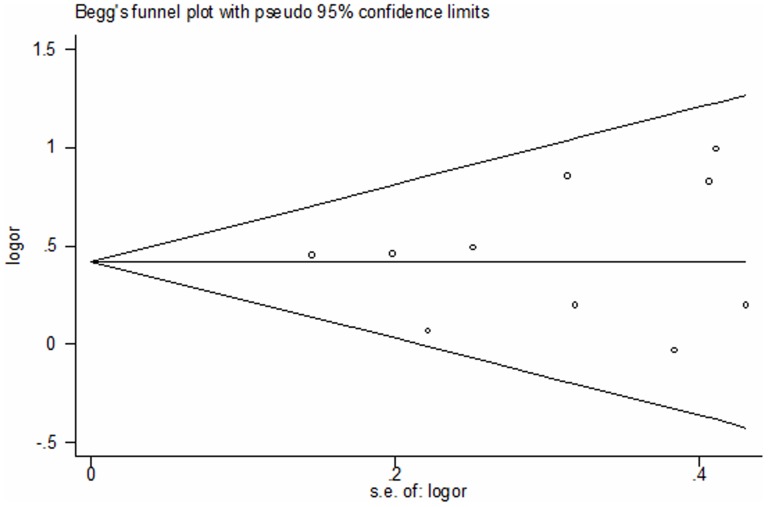
Funnel plot for publication bias of the meta-analysis of OSAS risk and *TNF-α-308G/A*polymorphism (A vs. G).

Begg's test and funnel plot were performed to verify the publication bias of the literature. The shapes of the funnel plots did not show obvious asymmetry (Figure 4). The asymmetry of the funnel plot was further tested by Egger's regression (t = 0.37, P = 0.724), which showed no significant publication bias.

## Discussion

In this meta-analysis, we concluded the existing data on the association of *TNFα-308G/A* polymorphism and OSAS risk. 10 studies involving 1,522 cases and 1,234 controls met the eligibility criteria. Our results demonstrated that the *TNF-α-308G/A* polymorphism was associated with OSAS risk (A *vs.* G: OR = 1.67, 95% CI = 1.43–1.95, P<0.00001). The A allele, compared with the G allele, was more likely to promote the risk of OSAS.

In stratified analyses, we observed that the association between *TNFα-308G/A* polymorphism and OSAS risk remained significant in Europeans (A *vs.* G: OR = 1.68, 95% CI = 1.35–2.08) and Asians (A *vs.* G: OR = 2.02, 95% CI = 1.50–2.71). Genetic background might have significant influence on the susceptibility of OSAS. However, up to date, no studies were performed in Africans, which limited our further analysis in those populations. At the same time, it was worth emphasizing that *TNFα-308G/A* polymorphism was contributed to the increase of OSAS susceptibility in adults (A *vs.* G: OR = 1.79, 95% CI = 1.50–2.13), but not for children (A *vs.* G: OR = 1.09, 95% CI = 0.70–1.69). This might reflect the fact that increased age modified the associations and might helpful to understand the pathogenesis of adults' OSAS.

The results from our study showed that *TNFα-308G/A* polymorphism associated with risk of OSAS. Compared with G allele, A allele was closely related to increased susceptibility to OSAS. The significance of this polymorphism could be explained by its possible influence on the expression of TNF-α protein. It was reported that the A allele of *TNF-α* gene increased TNF-α expression and resulted in higher TNF-α production by means of regulating adipose tissue metabolism [31], [32]. Moreover, TNF-*α* played an important role in regulating the pro-inflammatory cytokines during sleep and closely related to the level of high sensitivity C-reactive protein (hsCRP) [33]. Shamsuzzaman et al. demonstrated that the plasma C-reactive protein was elevated in OSAS patients and the magnitude of CRP elevation was remarkably related to the severity of OSAS [34].

Compared with the previous meta-analysis performed by Huang et al [35], our study had several superiorities. Firstly, the previous study identified less than half the number of studies as compared with the present study. More subjects were included in our study to provide a more comprehensive result. Secondly, in the previous meta-analysis, the authors did not evaluate some clinically significant outcomes and clinically relevant subgroups. This is first meta-analysis showed *TNF-α-308* polymorphism increased susceptibility to the OSAS in both in European and Asian population. In addition, we firstly observed *TNF-α-308* polymorphism increased the risk of OSAS in Adults, but not for children. Thirdly, no significant between-study heterogeneity and publication bias were observed in our study. Moreover, we carried out sensitivity analyses to evaluate the effect of each individual study on the pooled ORs.

Some limitations of this meta-analysis should be addressed. First, the number of published studies, especially for African population and children, was not large enough. Second, one study did not agreed with HWE in our analysis [26]. However, pooled ORs were not significant affect when the study was excluded. Third, in most studies detail information such as the co-morbidities were not available, which limited our further performed a risk-adjusted analysis.

## Conclusion

In summary, this meta-analysis suggested that the A allele of the *TNF-α-308* polymorphism increased susceptibility to the OSAS. These results provided the important role of the *TNF-α-308G/A* polymorphisms in the development of OSAS. Additional well-designed large studies were needed to validate our findings.

## Supporting Information

Checklist S1 (DOCX)

Table S1 **Quality assessment of case-control studies.**
(DOC)
