# Supplementary material for: TNF-α-308G/A Polymorphism Contributes to Obstructive Sleep Apnea Syndrome Risk: Evidence Based on 10 Case-Control Studies
Source: PLoS One. 2014 Sep 5;9(9):e106183. doi: 10.1371/journal.pone.0106183 (PMC4156295; doi:10.1371/journal.pone.0106183)
Supplement: Checklist S1 — (DOCX) [file pone.0106183.s001.docx]

**Meta-analysis on Genetic Association Studies Checklist | PLOS ONE**

|  | Item | Section name and paragraph number within manuscript |
| --- | --- | --- |
|  | **Introduction** |  |
| 1 | Provide a detailed justification for the polymorphism studied; if a single polymorphism was analyzed, give details as to why others were not included in the meta-analysis. | page:3-4  line:55-68 |
| 2 | Provide a detailed justification for the population(s) and clinical condition studied. | page:3  line:41-54 |
|  | **Methods** |  |
| 3 | Provide full details of the search strategy employed; outline the full electronic search strategy –specific combination of keywords and any limits applied- for at least one database. Specify whether synonyms of polymorphisms/genes (e.g. SNP number) were searched. | page:4  line:74-82 |
| 4 | Report full details on the inclusion and exclusion criteria applied for selecting studies.  *Please list the excluded articles and the reasons for exclusion of each article in a supplementary file.* | page:5  line:86-91 |
| 5 | Provide details on how the quality of the studies included in the analyses was assessed. | page:5  line:100-104 |
| 6 | Describe steps taken to contact study authors to identify additional studies and to request missing data. | N/A |
| 7 | Describe how environmental effects were adjusted for, if this adjustment was not conducted, outline the reasons for this. | N/A |
| 8 | Describe the methods of handling heterogeneity/between-study variance. | page:6  line:112-115 |
| 9 | Describe how the Hardy-Weinberg equilibrium and linkage disequilibrium were assessed. | page:6  line:108-109 |
| 10 | Describe and justify the choice of model for the analyses (per-allele vs per-genotype vs genetic model-free, random effects vs fixed effects). | page:6  line:116-119 |
| 11 | Describe whether a sensitivity analysis has been completed. | page:6  line:120-121 |
| 12 | Describe whether an assessment of the effects of population stratification has been conducted. | page:6  line:119-120 |
| 13 | Describe whether study-specific results have been assessed and if so the reasons for this (e.g. forest plot). | N/A |
|  | **Results** |  |
| 14 | Include flow diagram for the studies included in the meta-analysis as the first figure for the manuscript | page:6-7  line:126-131 |
| 15 | Report details on allele/genotype prevalence. | Table 1 |
| 16 | Report the effect size estimates and p values for each analysis. | page:7-8  line:146-158 |
|  | **Discussion** |  |
| 17 | Discuss the limitations of the meta-analysis, including genotyping errors/bias and publication bias. | page:10  line:206-209 |
| 18 | If the meta-analysis identifies an association within a subgroup of the population studied but not another, discuss the implications of these results, and if applicable the possibility of subgroup-specific publication bias. | page:9  line:179-188 |
| 19 | Discuss the suitability of the sample size employed to the research question and the power of the study. | page:9  line:173-178 |
