# Supplementary material for: TNF-α-308G/A Polymorphism Contributes to Obstructive Sleep Apnea Syndrome Risk: Evidence Based on 10 Case-Control Studies
Source: PLoS One. 2014 Sep 5;9(9):e106183. doi: 10.1371/journal.pone.0106183 (PMC4156295; doi:10.1371/journal.pone.0106183)
Supplement: Table S1 — Quality assessment of case-control studies. (DOC) [file pone.0106183.s002.doc]

| Supplementary table 1 Quality assessment of case-control studies | | | |  |  |  |  |  |  |
| --- | --- | --- | --- | --- | --- | --- | --- | --- | --- |
| **Reference** |  | **Selection** | |  | **Comparability** |  | **Outcome** |  | **Overall** |
|  | *Definition of cases* | *Representativeness of cases* | *Selection of controls* | *Definition of controls* | *Comparability the design or analysis* | *Ascertainment of exposure* | *Same method for cases and controls* | *Non-Response rate* | **Quality** |
| Popko et al, 2008^24^ | ★ | ★ | – | ★ | ★ | ★ | ★ | ★ | 7 |
| Khalyfa et a, 2011^22^ | ★ | ★ | ★ | ★ | ★ | ★ | ★ | ★ | 8 |
| Riha et al, 2005^27^ | ★ | ★ | ★ | ★ | – | ★ | ★ | ★ | 7 |
| Liu et al, 2006^25^ | ★ | ★ | ★ | ★ | ★ | ★ | ★ | ★ | 8 |
| Karkucak et a, 2012^20^ | ★ | ★ | ★ | ★ | ★ | ★ | ★ | ★ | 8 |
| Almpanidou et al, 2012^21^ | ★ | ★ | ★ | ★ | ★ | ★ | ★ | ★ | 8 |
| Li et al, 2013^18^ | ★ | ★ | – | ★ | – | ★ | ★ | ★ | 6 |
| Bhushan et al, 2009^23^ | ★ | ★ | ★ | ★ | ★ | ★ | ★ | ★ | 8 |
| Guan et al, 2013^19^ | ★ | ★ | ★ | ★ | ★ | ★ | ★ | ★ | 8 |
| Li et al, 2006^26^ | ★ | ★ | ★ | ★ | ★ | ★ | ★ | ★ | 8 |
